# Synthesis and Characterization of Pressure-Sensitive Adhesives Based on a Naphthyl Curing Agent

**DOI:** 10.3390/polym15234516

**Published:** 2023-11-24

**Authors:** Junhua Chen, Shiting Li, Xuan Wang, Lili Fang, Dingding Huang, Lin Ke, Jinlian Chen, Qingwei Wang, He Zhang, Yinping Wu, Dongyu Zhu, Chunsheng Li, Xiangying Hao

**Affiliations:** 1 School of Environmental and Chemical Engineering, Zhaoqing University, Zhaoqing 526061, China; cehjchen@yeah.net (J.C.); lishitingqc@163.com (S.L.); pearships@163.com (X.W.); lily_ffa@163.com (L.F.); huangdingding1116@163.com (D.H.); kelin0515@163.com (L.K.); cjlccc2003@outlook.com (J.C.); wangqingwei0721@163.com (Q.W.); zhanghe@zqu.edu.cn (H.Z.); wuyinping@zqu.edu.cn (Y.W.); 2 Guangdong Provincial Key Laboratory of Environmental Health and Land Resource, College of Environmental and Chemical Engineering, Zhaoqing University, Zhaoqing 526061, China; 3 School of Chemical Engineering and Light Industry, Guangdong University of Technology, Guangzhou 510006, China; zdy16@gdut.edu.cn

**Keywords:** pressure-sensitive adhesives, thermal analysis, glass transition temperature, UV curing

## Abstract

The incorporation of a naphthyl curing agent (NCA) can enhance the thermal stability of pressure-sensitive adhesives (PSAs). In this study, a PSA matrix was synthesized using a solution polymerization process and consisted of butyl acrylate, acrylic acid, and an ethyl acrylate within an acrylic copolymer. Benzoyl peroxide was used as an initiator during the synthesis. To facilitate the UV curing of the solvent-borne PSAs, glycidyl methacrylate was added to introduce unsaturated carbon double bonds. The resulting UV-curable acrylic PSA tapes exhibited longer holding times at high temperatures (150 °C) compared to uncross-linked PSA tapes, without leaving any residues on the substrate surface. The thermal stability of the PSA was further enhanced by adding more NCA and increasing the UV dosage. This may be attributed to the formation of cross-linking networks within the polymer matrix at higher doses. The researchers successfully balanced the adhesion performance and thermal stability by modifying the amount of NCA and UV radiation, despite the peel strength declining and the holding duration shortening. This research also investigated the effects of cross-linking density on gel content, molecular weight, glass transition temperature, and other properties of the PSAs.

## 1. Introduction

Pressure-sensitive adhesives (PSAs) are capable of adhering to solid substrates quickly and with minimal pressure, and they can be easily removed without leaving any visible residue [1,2,3]. Due to their viscoelastic nature, PSAs exhibit semi-solid properties that can be evaluated through measurements of tack, peel resistance, and shear strength [4]. Acrylic PSAs are commonly employed in various industrial products, including mounting tapes, packing tapes, labels, and protective films [5]. One specific application of PSAs involves their use as a protective coating for electronic chemical materials [6,7,8]. The protective PSA film serves to safeguard the electronic components from external factors. The rapid advancement of integrated circuit technology necessitates the incorporation of thermal stability and enhanced reliability in the protective PSA films for microdevices [9].

The primary constituents employed in the production of PSAs through solution and emulsion polymerization methods are commonly acrylic ester monomers. Nevertheless, the inherent linear structures of the polymer constituents in synthetic PSAs impose constraints on their tensile and thermal properties [10]. In order to enhance the thermal resilience of PSAs, it becomes imperative to restrict the mobility of polymeric chains. In general, modulating the degree of cross-linking in PSAs represents a highly effective approach for minimizing molecular motion. UV curing is a widely employed cross-linking method for PSAs due to its numerous advantages, including environmentally friendly emissions, reduced power consumption, and accelerated curing speed [11,12,13,14]. The UV-curable systems used to cross-link acrylic PSAs are made up of a photoinitiator and unit with unsaturated carbon double bonds that help with subsequent cross-linking [9,15,16].

The utilization of the aromatic compound containing naphthyl groups in materials is facilitated by its thermal resistance and protective packaging capabilities [17]. This compound exhibits a higher glass transition temperature (T_g_) and enhanced thermal stability. Previous studies have demonstrated that the introduction of rigid structures, such as the naphthyl group, into the molecular chains can effectively enhance thermal stability [9,18,19,20]. The UV-curable, acrylic pressure-sensitive adhesives (PSAs) possess a cross-linked network structure comprising acrylic polymer links and a curing-agent component [21]. The adhesive properties of the acrylic component are closely associated with its flexibility. Furthermore, it has been observed that the molecular structure of UV-curable, acrylic PSA is not affected by the curing agent [7,22]. Therefore, incorporating the naphthyl group into the polymeric chains is a viable method for obtaining a thermally stable structure. This modification results in a higher T_g_, better thermal degradation and flame retardancy, and increased carbon residue compared to acrylate PSAs. Despite the benefits observed, there has been a lack of reports on the utilization of the naphthyl group for enhancing the thermal stability of PSAs.

Thermal stability is a key property of PSAs in electronic chemical materials such as packaging and protection. To improve the thermal stability of adhesion, PSAs were synthesized using an NCA in this study. The UV-curable, acrylic PSAs were then cross-linked to enhance their mechanical, adhesive, and thermal properties. The impact of the amount of NCA and UV radiation on these properties were carefully analyzed. These results demonstrate the potential of an NCA and UV irradiation in enhancing the thermal stability of PSAs, a crucial factor in electronic applications. This research is highly relevant to peers and researchers working in related fields.

## 2. Materials and Methods

### 2.1. Materials

The monomers of n-butyl acrylate (BA), acrylic acid (AA), and vinyl acetate (vAc) of chemical purity were procured from Shanghai Aladdin Reagents Co., Ltd. Benzoyl peroxide (BPO, industrial grade, Shanghai Aladdin Reagents Co., Ltd., Shanghai, China) and ethyl acetate (EAC, industrial grade, Guangzhou Chemical Reagent Factory, Guangzhou, China) were utilized as the radical initiator and solvent in radical polymerization. The acrylic copolymer was modified using glycidyl methacrylate (GMA, industrial grade, Shanghai Aladdin Reagents Co., Ltd., Shanghai, China), and the reactive vinyl group was retained. The naphthyl curing agent was synthesized using 1,5-dihydroxynaphthalene (DHNA, industrial grade), 4-dimethylaminopyridine (DMAP), and acryloyl chloride (chemical pure), which were all procured from Shanghai Aladdin Reagents Co., Ltd. 1-Hydroxy-cyclohexyl-phenyl ketone (CP-4, industrial grade, Tianjin Damao Chemical Reagent Co., Ltd., Tianjin, China) was employed as a photoinitiator. All the other reagents underwent further purification before use.

### 2.2. Preparation of Acrylic Copolymer

A polymerization process was performed to produce an acrylic copolymer containing butyl acrylate (BA, 80 g), vinyl acetate (Vac, 15 g), and acrylic acid (AA, 5 g). The monomers were combined with 2 g of benzoyl peroxide (BPO) in 185 mL of ethyl acetate. The solvent-borne acrylic copolymer was synthesized in a 500 mL flask equipped with a condenser, mechanical stirrer, and dropping funnel, with the polymerization carried out at 80 °C for a dosage time of 3 h, followed by post-reaction at 80 °C for an additional 3 h. The acrylic resin was subsequently reacted with glycidyl methacrylate (GMA) at a temperature of 50 °C for a period of 12 h, resulting in the synthesis of UV-curable acrylic copolymer. The number-average molecular weight of the acrylic copolymer using gel permeation chromatography was determined to be 1.0 × 10^5^, with a polydispersity value of 3.23.

### 2.3. Synthesis of the NCA

**Scheme 1:**
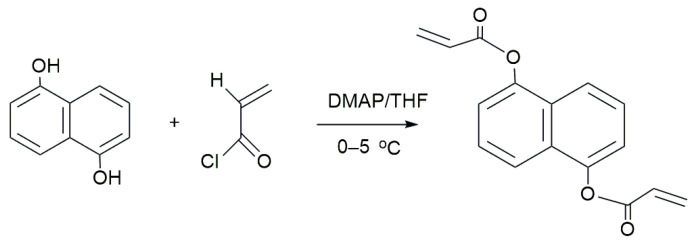
Synthesis of the NCA.

In our laboratory, the NCA was synthesized through a one-step process as depicted in Scheme 1. The procedure involved adding 20 g of DHNA, 40 mL of triethylamine, 0.5 g of 4-dimethylamineopyridine, 0.5 g of hydroquinone, and 100 mL of tetrahydrofuran into a 250 mL single-neck, round-bottomed flask which was equipped with a thermometer, stirrer, and dropping funnel. Meanwhile, acryloyl chloride (38 mL) dissolved in tetrahydrofuran (50 mL) was slowly added while stirring, maintaining the temperature at 0–5 °C. The mixture was then stirred for 4 h at room temperature and subsequently filtered. The undesired by-products were eliminated by treating the reaction mixture with a 10% dilute hydrochloric acid solution, followed by 200 mL of distilled water, and a 5% potassium carbonate solution. The crude product was filtered, washed with ethanol, and dried. Ultimately, a product with a yield of 53% was obtained after the filtration and removal of ethanol [23].

### 2.4. Preparation of the UV-Curable, Acrylic PSA Film

**Scheme 2:**
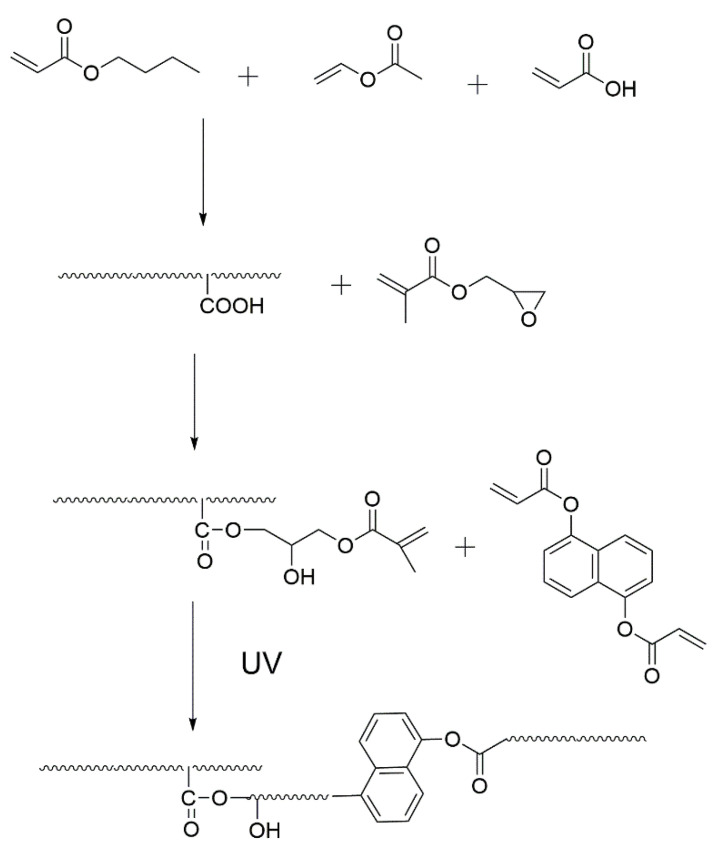
Preparation of UV-curable, acrylic PSAs.

**Scheme 3:**
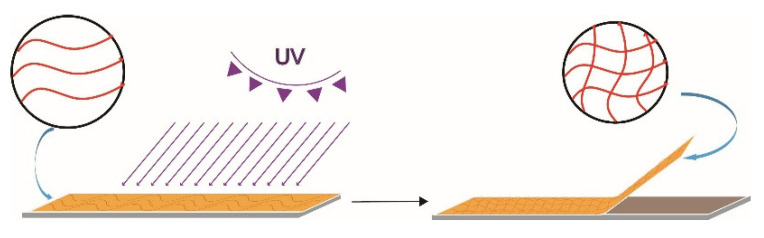
Illustration of the UV-curable PSAs.

The acrylic copolymer solution was supplemented with an NCA of 0–20 phr to PSA and CP-4 (5 wt% to PSA). The UV-curable, acrylic PSA was prepared by mechanically stirring the solution at room temperature. The process for creating the UV-curable, acrylic PSA resin is displayed in Scheme 2 and Scheme 3. The resulting mixture was applied onto a 90 μm thick poly(ethylene terephthalate) (PET) film using an applicator roll, ensuring a uniform coating thickness. The coated surface was then dried in an air drying oven at 120 °C for 5 min to eliminate the solvent. As a result, the acrylic PSA layer had a thickness of approximately 20 ± 3 μm using a coating thickness gauge. Finally, the acrylic PSA films were cross-linked through UV-irradiation, utilizing commercially available UV lamps.

### 2.5. Characterization

We utilized an FT-IR analyzer (MAGNA-IR750, Nicolet, Madison, WI, USA) to acquire the Fourier-transform infrared (FT-IR) spectra of the NCA and the resulting reaction products within the frequency range of 4000–400 cm^−1^. Meanwhile, ^1^H-NMR of the NCA was captured on a Bruker AVANCE AV400 analyzer (Romanshorn, Switzerland) at room temperature, utilizing CDCl_3_ as a solvent and TMS as an internal reference. After undergoing the cross-linking and UV-curing processes, the PSA polymers were synthesized. Before immersing in ethyl acetate at 60 °C for 72 h, the gel contents of the PSA samples were estimated. Subsequently, a 200-mesh-wire filtration was performed on the samples, followed by drying until a consistent weight was attained. The gel concentration of the UV-curable, acrylic PSA samples was determined utilizing the subsequent algorithm (1):

*Gel content* (%) = (*W_t_*/*W_0_*) × 100 (1)

where *W_t_* is the weight of the dried sample, and *W_0_* is the initial weight of the sample.

The molecular weights of the sticky polymer solutions were measured using gel permeation chromatography (Malvern, UK). To dissolve a small amount of dry adhesive, tetrahydrofuran (THF) was utilized, and the mixture was shaken for 24 h. The resulting solution was then purified using a 0.2 μm polytetrafluoroethylene filter. The injection volume was 0.1 mL, and the mobile-phase flow rate was 1 mL/min. Glass transition temperatures (T_g_) were measured using a DSC 200 F3 instrument. Desiccated samples weighing 5 mg were packed into metal sample pans. The samples were cooled to −60 °C for 3 min before being heated to 120 °C at a rate of 10 °C/min.

Based on ASTM D3121-17 (Standard Test Method for Tack of Pressure-Sensitive Adhesives by Rolling Ball), PSA tapes with a cross-section of 100 mm × 100 mm were tested for tack using a tester (TSKL-CNS). The adhesive was applied to stainless-steel pans meticulously wiped with tissue and measured 25 mm wide. The maximum number of sizable spheres adhering to the adhesive during the descent of varied-size spheres over a sloping surface underneath it was quantified. The 180° peel strength of PSA tape was tested according to ASTM D1000-66. The adhesive tapes underwent reciprocation thrice by a traditional roller. Electronic tensile testing equipment (MIDEL KJ-1065A, Dongguan Kejian Testing Instrument Co., Ltd., Dongguan, China) operating at a velocity of 300 mm/min gauged the 180° peel strength post 30 min. The cohesive strength of PSA tapes was tested using a lasting adhesive tester (MIDELKJ-6032, Dongguan Kejian Testing Instrument Co., Ltd., Dongguan, China) according to ASTMD 3653. The stainless-steel carriers were bedded with standardized tapes measuring 25 mm × 70 mm. A customary roller facilitated reciprocation thrice for enhanced surface interaction between the PSA adhesive and the substratum. The specimens were subsequently elevated upright within the testing apparatus one hour later with a mass load of 2 kg. The pace at which the adhesive detached from the steel sheets was employed to compute the shear anchoring capacity. The temperature holding force tester (MIDEL KJ-6012, Dongguan Kejian Testing Instrument Co., Ltd., Dongguan, China) was set to 100 °C and 150 °C, which was the same as the cohesive strength of the PSA tapes, so as to evaluate the high-temperature shear resistance of the pressure-sensitive adhesive.

A Perkin-Elmer Pyris-1 TG Analyzer (Waltham, WI, USA) was leveraged to assess the thermal stability of the PSA specimens. Under nitrogen conditions, the samples underwent heating from 25 °C to 600 °C at a gradient of 10 °C/min. For another 85 min, the samples were maintained at 300 °C inside a nitrogen environment to check their isothermal durability. Cotton immersed in ethanol-derived solvent was used to clean the stainless-steel platform. Three imprints using a 2 kg roller were made on the adhesive tape samples affixed to the steel pans. After this, the adhesive tape specimens were subjected to four hours of intense heat at 150 °C in an oven. As soon as the tapes reached room temperature, the interfaces of the stainless-steel planes and the residue remnants were meticulously scrutinized. The detachment velocity from the steel sheets was calculated to determine the shear adhesiveness. The experimental procedure remained consistent with the standard shear resilience examination, the sole deviation being the increased temperature (150 °C), accomplished through the oven placement of the test specimens.

## 3. Results and Discussion

**Figure 1:**
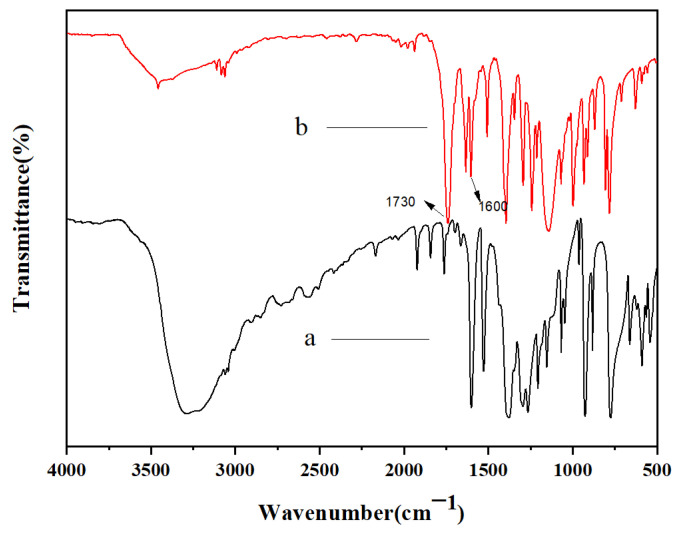
FTIR spectra of DHNA (**a**) and the NCA (**b**).

**Figure 2:**
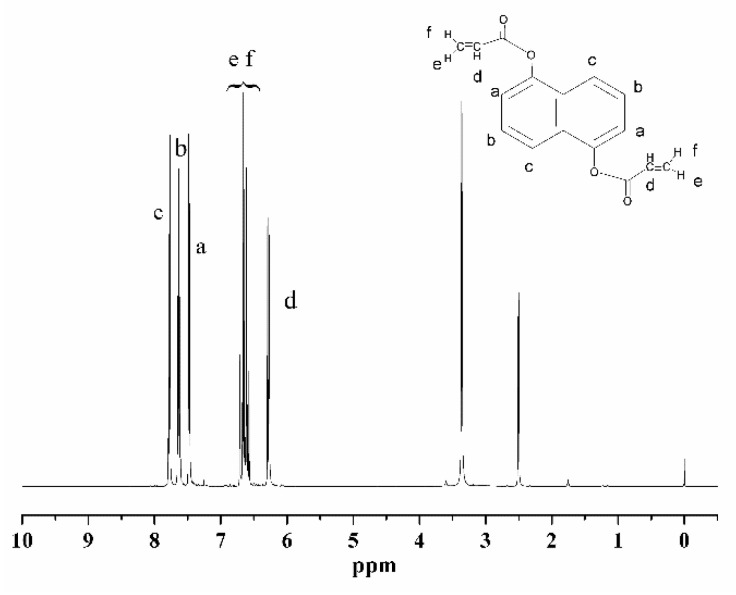
^1^H-NMR spectra of the NCA.

Figure 1 illustrates the FTIR spectra of DHNA and the NCA, where Line a represents DHNA and Line b represents the curing agent. In Line b, the significant peaks at 1250–1200 cm^−1^ and 1730 cm^−1^ corresponded to the C-O and C=O groups, respectively. Additionally, a distinctive absorption band of the C=C group was seen at 1590–1640 cm^−1^, and the peak of the -OH group around 3000–3500 cm^−1^ disappeared in Line b, indicating the presence of C=C groups in the monomer of the curing agent. Figure 2 displays the ^1^H-NMR spectrum of the NCA (with TMS as the standard and CDCl_3_ as the solvent). The spectrum reveals the expected peaks, including 6.27 (-CH attached to the monomer chains), 6.59–6.71 (=CH_2_ attached to the -C=C side group of the monomer), and 7.46–7.78 (-CH attached to the naphthyl group of the monomer). The signals at 2.5 and 3.3 ppm potentially indicate the presence of a hydroxyl structure with a small amount of residual DNHA. Both the FTIR and ^1^H-NMR spectra suggest the completion of the reactions and the successful synthesis of the expected monomer.

**Figure 3:**
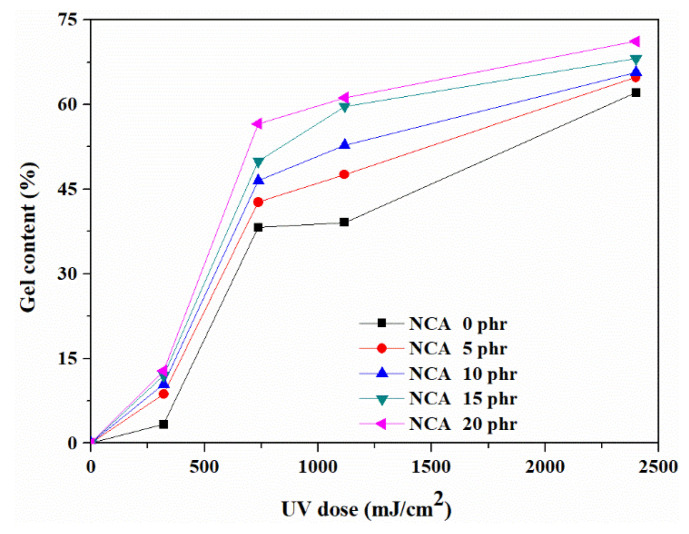
Variations in the gel content of PSA composites depending on NCA content and UV dose.

The determination of gel fraction is a useful method for assessing the degree of cross-linking in cross-linked or network polymers [24,25]. Figure 3 illustrates the gel content of the modified pressure-sensitive adhesives (PSAs) under UV irradiation with varying amounts of NCA and at different UV doses. The gel fraction offers insight into the amount of cross-linking in the system and better explains the impact of UV doses and NCA contents on the degree of cross-linking. While both chain scission and cross-linking can occur simultaneously following UV irradiation, we evaluate the outcome of such treatment. The gel content of PSAs increases gradually with increasing amounts of NCA, which is due to the high concentration of carbon double bonds in the composites that can form cross-links with acrylic PSAs [26]. When the NCA amount was 15 phr, the gel content reached 68% at the UV dose of 2400 mJ/cm^2^. The gel content also increased with increasing UV doses, due to the enhanced potential for cross-linking between the acrylic copolymer and NCA. This cross-linking density was able to increase with the higher UV dose. Due to the tight interlocking of the unsaturated linear chain segments, the highly cross-linked network structure changed numerous PSA characteristics, including its mechanical, thermal, and adhesive capabilities [27].

**Figure 4:**
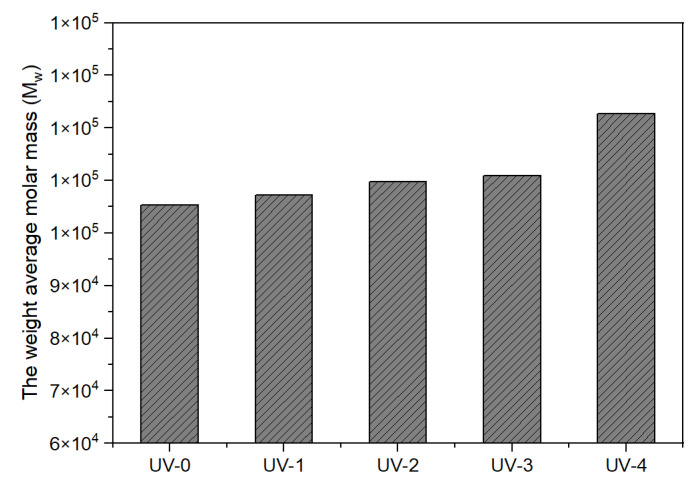
Effect of UV dose on *M_w_* (the amount of addition: 10 phr).

**Figure 5:**
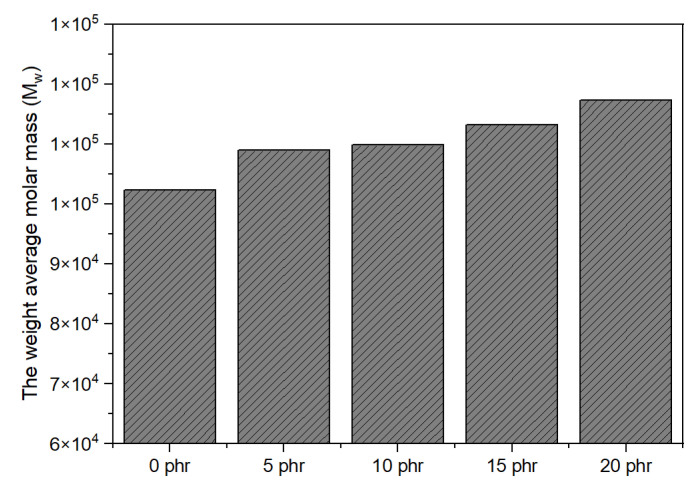
Effect of NCA concentration on *M_w_* (UV dose: 737 mJ/cm^2^).

Figure 4 demonstrates the influence of various UV doses on the weight average molar mass (M_w_) of composites with 10 phr NCA. The corresponding groups of UV dose were 0 mJ/cm^2^ for UV-0, 320 mJ/cm^2^ for UV-1, 737 mJ/cm^2^ for UV-2, 1116 mJ/cm^2^ for UV-3, and 2400 mJ/cm^2^ for UV-4. The M_w_ was observed to increase as the UV dose increased. This phenomenon can be attributed to the initiation of radical polymerization reactions among the C=C bands in the composites, initiated by the radicals generated by the photoinitiators through UV irradiation. This ultimately resulted in a highly cross-linked structure, leading to a higher M_w_. Figure 5, on the other side, shows the impact of NCA concentration on Mw when the UV dose remained constant. M_w_ has been identified to rise with increasing NCA content. This suggests that during polymerization, there were more cross-linking points available, allowing longer polymer chains to form. In summary, the concentration of the NCA and the UV dose played a significant role in the trend of the M_w_, which exhibited a rapid increase as the concentration of the NCA and UV dose increased.

**Figure 6:**
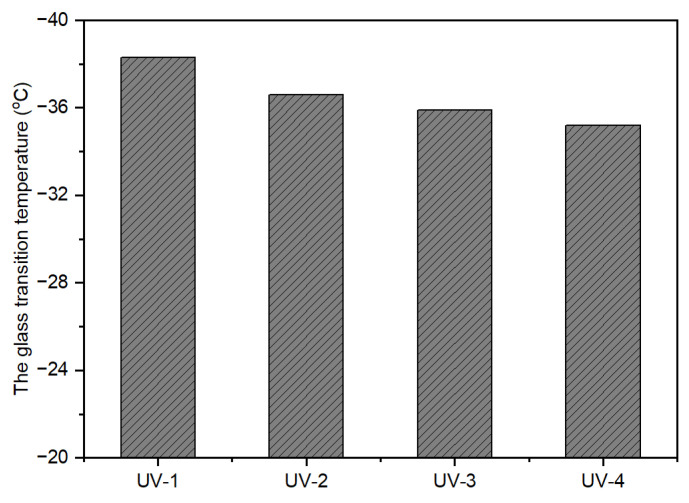
Effect of UV dose on T_g_ (the amount of addition: 10 phr).

**Figure 7:**
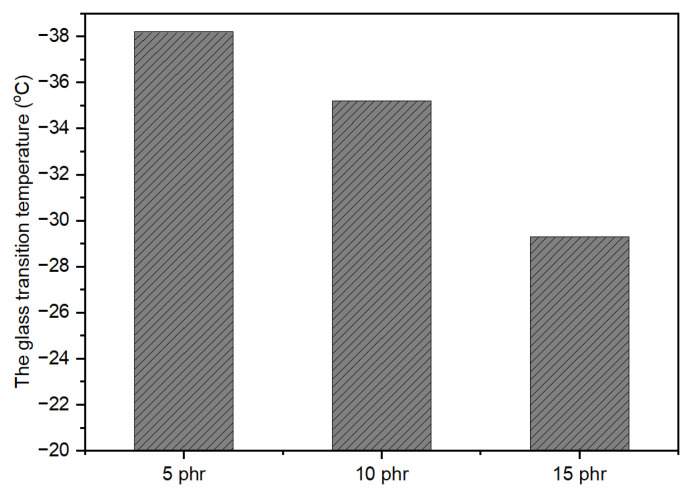
Effect of NCA amount on T_g_ (UV-4: 2100 mJ/cm^2^).

The calculation of glass transition temperature (T_g_) through the Fox equation (Equation (2)) for uncross-linked, UV-curable, acrylic pressure-sensitive adhesives (PSAs) resulted in a T_g_ close to −40 °C. However, the actual measured value was higher than the calculated value. One plausible explanation for this discrepancy is the formation of hydrogen bonds between the polar carboxyl groups in the polymer chains, contributing to physical cross-linking [1]. As observed in Figure 6, the T_g_ value increased as UV dose increased when the cross-linker content was at 10 phr. This can be attributed to the highly cross-linked networks formed in the polymer chains due to the greater extent of UV-induced cross-linking reactions, which can hinder the movement of molecular chains. Furthermore, Figure 7 shows that the T_g_ increased with an increase in the amount of NCA at a UV dose of 2100 mJ/cm^2^.

The T_g_ of a polymer is influenced by various factors, including comonomers present in the reaction mixture. The T_g_ plays a critical role in determining the softness of the acrylic PSAs, which is necessary for flow and adhesion to surfaces [28]. Typically, the using comonomers like Vac and AA increase the T_g_ of the adhesive, enhancing its cohesive strength. The introduction of an NCA with a high T_g_ into the monomer mixture is expected to modify the T_g_ of the polymer.

(2) $$\frac{1}{T_{g}}=\frac{w_{1}}{T_{g}1}+\frac{w_{2}}{T_{g}2}+\cdots$$

where T_g_ is the glass transition temperature of the copolymer, T_g_1 and T_g_2 are the glass transition temperatures of Component 1 and Component 2 in the copolymer, and ω_1_ and ω_2_ are the mass fractions of Component 1 and Component 2, respectively.

In summary, the physical and chemical properties of the polymer, including T_g_, are vital considerations in the design and preparation of PSAs. The information presented in this paper sheds light on the factors that affect T_g_ and their implications on the adhesion properties of PSAs. Understanding these underlying mechanisms can facilitate the development of PSAs with improved performance in various industrial and biomedical applications.

**Figure 8:**
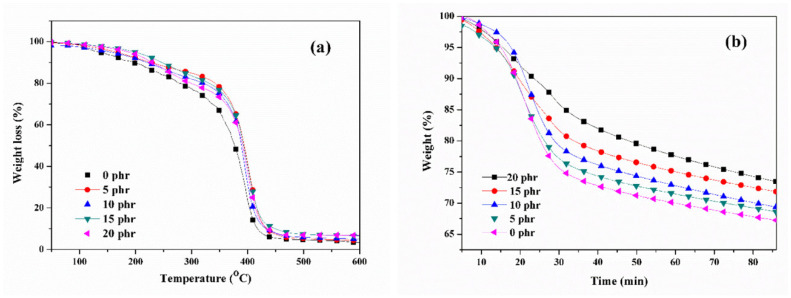
TGA thermograms of PSAs with various NCA content in a nitrogen atmosphere after irradiation with a UV dose of 2100 mJ/cm^2^: (**a**) heating from 30 °C to 500 °C at 10 °C/min; (**b**) isothermally kept at 300 °C for 85 min.

In order to assess the impact of the naphthyl curing agent on the thermal stability of acrylic pressure-sensitive adhesives (PSAs), the thermal properties of UV-curable, acrylic PSAs were analyzed using TGA. The TGA thermograms of the UV-curable, acrylic PSAs with varying amounts of NCA content in a nitrogen atmosphere, after irradiation with a UV dose of 2100 mJ/cm^2^, are presented in Figure 8a. The primary breakdown of the PSA samples started between 200 °C and 380 °C due to the thermal degradation of the polymer matrix [29]. The temperatures of 60% weight loss and residue content at 600 °C are presented in Table 1. The samples exhibited similar degradation trends. In comparison to the samples cross-linked without the NCA, the residual concentration and decomposition temperature of the UV-curable PSA samples significantly increased. This suggested that the enhancement of thermal stability could be attributed to the expanding cross-linking density of the films [30]. The major thermal dissociation temperature of the UV-curable, acrylic PSAs increased, primarily due to stronger covalent connections formed between the copolymer and NCA. These connections introduced the thermally stable aromatic unit to the main chains of PSAs. This incorporation of rigid structures into the acrylic PSAs was accomplished through the resonance structures of aromatic groups [31]. The introduction of aromatics was a simple and effective method for improving thermal stability. Figure 8b presents the weight loss curves of the UV-curable, acrylic PSA samples, cross-linked and kept at 300 °C for 85 min, after irradiation with a UV dose of 2100 mJ/cm^2^. The isothermal stability of the samples at 300 °C was also significantly improved with increasing NCA content. This was attributed to the contribution of the thermally stable naphthyl structure introduced to the acrylic chains of the PSAs, as well as the rigid network structures formed between the acrylic copolymer and NCA.

**Figure 9:**
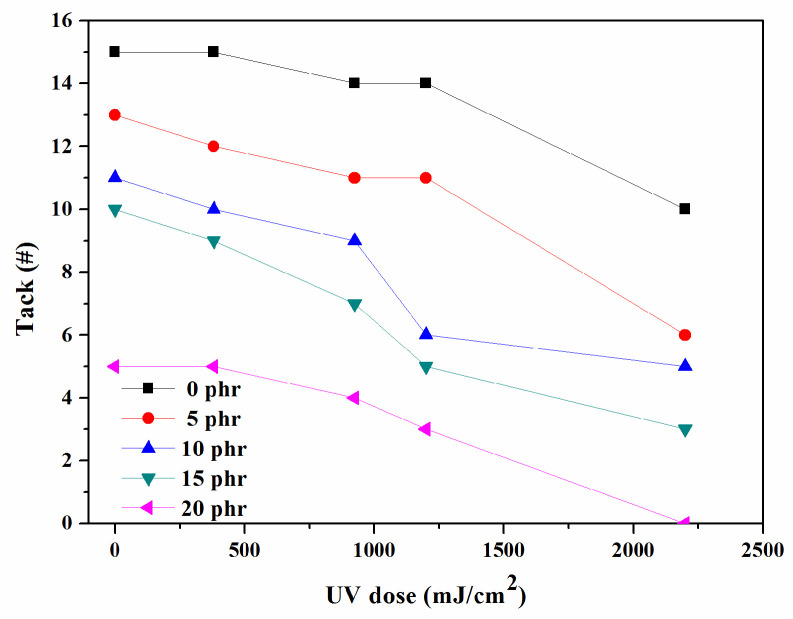
Variations in the tack of PSAs.

The adhesive attribute known as “initial tack” is measured to assess the effectiveness of PSAs, with the highest number of the largest balls that remain on the PSA surface recorded. Figure 9 illustrates the variation in tack for UV-curable, acrylic PSAs with different NCA contents and UV doses. As the NCA and UV radiation increased, the tack decreased. Given that tack is a crucial adhesion feature that allows materials to stick to substrate surfaces quickly and with minimal pressure, it is likely that it heavily relies on the mobility of molecular chains. The cross-linking parts, which decrease the mobility of molecular chains, lead to a decrease in the tack of UV-curable, acrylic PSAs. This reduction in tack can prevent the substrate from becoming wet on the surface and reduces the contact surface of the tapes with the substrate. Therefore, improving the mobility of polymer chains could enhance the efficacy of the adhesion properties of UV-curable, acrylic PSAs.

**Figure 10:**
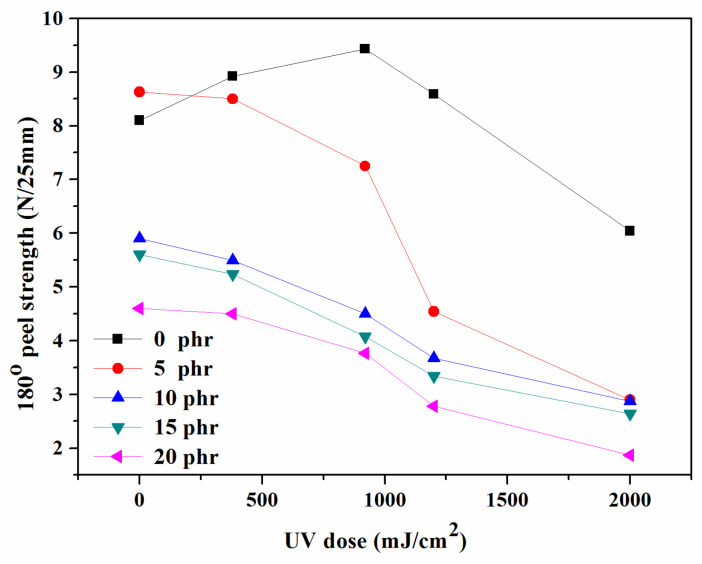
Variations in the peel strength of acrylic PSAs.

The peel strength was frequently the main consideration while analyzing the properties of PSAs. The peel strength exhibited a similar behavior to the tack, indicating that the mobility of the polymeric molecules in acrylic PSAs played a significant role in determining the peel strength. Several studies investigated the correlation between increasing PSA cross-linking levels and peel strength [32,33,34,35]. During the peel strength test, the critical features of the PSAs resulted in interfacial failure, influenced by the structure and cross-linking of the copolymer. Figure 10 shows that when the UV dose and NCA content increased, the peel strength of the PSAs considerably reduced. When the concentration of the NCA was 0 phr, the peel strength initially increased before declining. By utilizing UV light to cross-link the copolymer and the NCA, acrylic PSAs formed a complex network structure that severely limited the mobility of their polymeric chains [9]. The reduced peel strength can be primarily attributed to the restricted interfacial contact between the substrate and PSA tapes, as increased cross-linking further limits the mobility of the polymeric chains [9,18].

**Figure 11:**
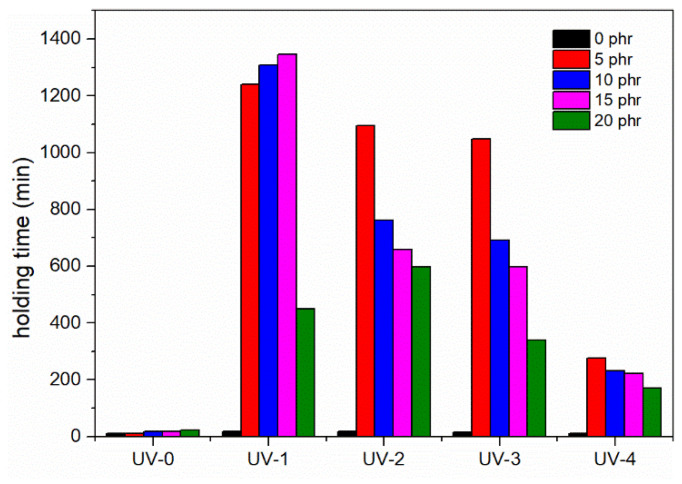
Variations in the holding time of acrylic PSAs (the unit of time: min; reaction temperature: 25 °C).

Figure 11 shows the shear strength of UV-curable, acrylic pressure-sensitive adhesives (PSAs) containing the NCA as a cross-linker, which was measured at room temperature. The results indicate that the shear strength of the UV-curable, acrylic PSAs was significantly affected by the amount of NCA and UV dosage used in the cross-linking process. Specifically, when the UV dosage was 380 mJ/cm^2^, the holding period increased from 19 min to 1347 min with an increase in NCA content, after which the holding time rapidly declined to 449 min. Similarly, the holding time initially increased and then decreased with increasing UV dosage when the NCA content was kept constant [10]. The observed results could be attributed to the formation of polymeric networks with interconnected macromolecules and cross-linking of the linear molecular chains of the PSAs with a small quantity of NCA and UV radiation. With an increase in the cross-linker and UV dosage, the number of cross-linking sites increased, leading to the enhanced cohesiveness of the adhesives. When the NCA content reached 5 phr or the UV dose was over 380 mJ/cm^2^, a highly cross-linked network structure was speculated to form. However, excessive cross-linking points could have a negative impact on the bonding, adhesive properties of PSAs.

**Figure 12:**
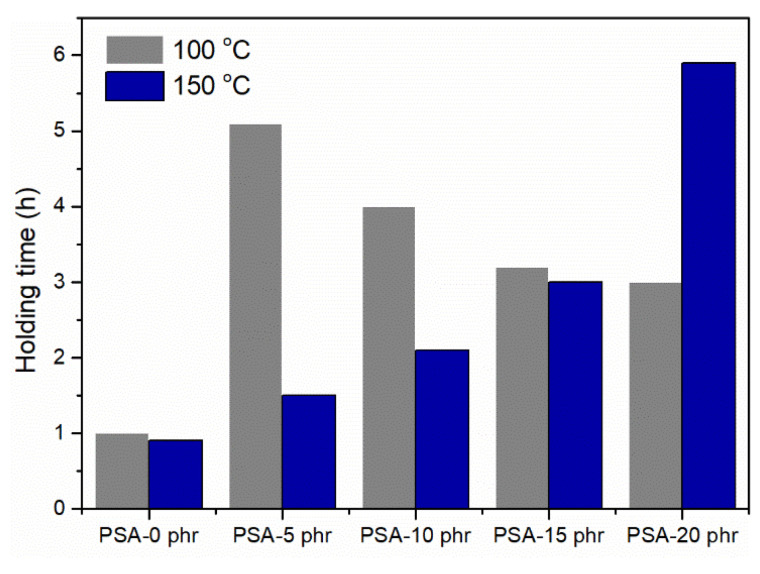
Effect of NCA content on the holding time at various temperatures.

As demonstrated in Figure 12, the shear strength of UV-curable, acrylic PSAs increased with a higher concentration of NCA at an elevated temperature (150 °C) after UV irradiation (UV dose: 2000 mJ/cm^2^). Conversely, when the NCA content exceeded 5 phr at 100 °C after irradiation, the shear strength decreased. This decline was attributed to an increased holding time caused by the creation of a cross-linking network. When the NCA was added in excess of 5 phr at 100 °C, the abundance of cross-linking points containing unsaturated bonds led to cohesion failure during high-temperature tests after a 2000 mJ/cm^2^ dosage of UV exposure. Notably, when the NCA content was 20 phr, the shear strength remained constant at 150 °C for approximately 6 min, indicating a significant improvement compared to when there was no NCA present. Additionally, the average molecular weight may have an impact on cohesive strength at the cross-linking locations (M_c_), which could be determined using Equation (3) as outlined in [36].

(3) $$M_{c}=\frac{V_{1}\rho \left[\varphi^{1/3}-\varphi /2\right]}{-\left[\ln\left(1-\varphi \right)+\varphi +\chi \varphi^{2}\right]}$$

*V*_1_: molar volume of toluene, *ρ*: density of the polymer, *φ*: volume fraction of the polymer in toluene, *χ*: polymer–solvent interaction parameter, *M_e_*: the theoretical average molecular weight of the network chain.

A substantial cross-linking network can be created by the linear acrylic PSAs when M_c_ ≥ M_e_ [37]. Increasing the amount of cross-linker enhances the cohesive strength and cross-linking density of the PSAs while reducing M_c._ This demonstrates an improvement in the properties and potential applications of the PSAs.

**Figure 13:**
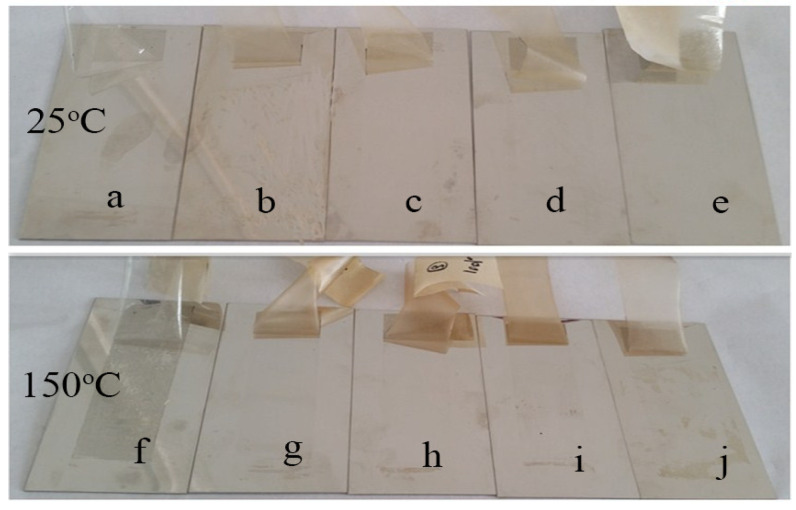
The thermoresistance of the tapes made of PSA cross-linked by 0 phr, 5 phr, 10 phr, 15 phr, and 20 phr of NCA were tested after UV exposure at 25 °C (**a**–**e**) and 150 °C (**f**–**j**).

Figure 13 demonstrates the effect of NCA content on the thermoresistance properties of cross-linked PSAs after a 2000 mJ/cm^2^ dosage of UV exposure. These PSAs had excellent peeling properties and left no residues on the surface of tape at room temperature. However, when the NCA content was 0 phr and the tapes were peeled at 150 °C, some evident residues were noticeable. This indicates that the degree of cross-linking was inadequate, leading to a low cohesive strength in the tapes, which resulted in ineffective debonding from the surface of steel sheets. Moreover, the adhesives were fluid, causing them to leave visible residues on the surface of the steel sheets when exposed to high temperatures. To overcome this challenge, we introduced rigid bonds to the polymeric molecules of the acrylic PSAs using UV irradiation, which subsequently underwent chemical cross-linking. The cohesive strength of the PSAs would increase during drying and UV irradiation, making them less likely to leave residues on the surface of the steel sheets. Figure 13f–j reveals that the peel properties of PSA tapes improved significantly at 150 °C. Compared to other cross-linkers, the appearance of these tapes left no residue on the surface of steel sheets, while maintaining excellent mechanical and thermal properties. Table 2 summarizes the results indicating that the thermoresistance test demonstrated that the UV-curable, acrylic PSAs had high stability at high temperatures. The incorporation of the NCA contributed to the stable configurations of the PSAs, thereby achieving superior thermoresistance performance.

## 4. Conclusions

In this study, vinyl groups added to an acrylic copolymer by GMA modification are used to create UV-curable PSAs, as well as an NCA which introduces thermally stable aromatic structures to the main chains. The addition of the NCA and a higher UV dose was found to increase the gel fraction of the PSAs due to the formation of numerous cross-links between the components. The concentration of the NCA was identified as the main contributor to the thermal stability of PSAs, as it effectively added stiff groups and created cross-linked structures in the polymer matrix. Consequently, the T_g_ of PSAs was significantly improved, leading to increased thermal stability. However, the addition of the NCA also resulted in decreased tack and 180° peel strength due to limitations in polymeric chain mobility. Nonetheless, the holding duration of the PSAs after UV irradiation was significantly improved due to the cohesive strength created by the cross-linked network. Furthermore, the use of the NCA allowed for the creation of PSAs with a range of applications in electronic chemical products, such as packaging and protection. These PSAs exhibited excellent adhesive properties at elevated temperatures, thanks to the formation of cross-linked networks between the acrylic polymer matrix and the NCA. Overall, this study demonstrates the potential of using a thermally stable NCA to enhance the properties of UV-curable, acrylic PSAs, enabling their use in a diverse range of applications.

## Figures and Tables

**Table 1:** Thermal resistance of UV-curable PSAs with a UV dose of 2100 mJ/cm^2^.

Samples	Corresponding Temperature for 60% Weight Loss (°C)	Residue Content (wt%)
0 phr	387	3.4
5 phr	399	4.3
10 phr	398	5.1
15 phr	399	6.6
20 phr	398	6.9

**Table 2:** The influence of cross-linker content on the appearance of the substrate at 24 h/150 °C (additive: 20 phr).

Samples	Appearance
Polyfunctional aziridine	Large residue
2,4-tolylene diisocyanate (TDI)	Small residue
GMA	Large residue
MQ silicone resin	No residue
NCA	No residue

## Data Availability

The data presented in this study are available on request from the corresponding author.
